# Adaptation and psychometric evaluation of the breastfeeding self-efficacy scale to assess exclusive breastfeeding

**DOI:** 10.1186/s12884-019-2217-7

**Published:** 2019-02-18

**Authors:** Godfred O. Boateng, Stephanie L. Martin, Emily L. Tuthill, Shalean M. Collins, Cindy-Lee Dennis, Barnabas K. Natamba, Sera L. Young

**Affiliations:** 1 Department of Anthropology & Global Health, Northwestern University, 1810 Hinman Avenue, Evanston, IL 60208 USA; 2 Department of Nutrition, Harvard T.H. Chan School of Public Health, Boston, MA USA; 3 Department of Nutrition, Gillings School of Global Public Health, University of North Carolina at Chapel Hill, CB 7461, Chapel Hill, NC 27599 USA; 4 Department of Community Health Systems, University of California San Francisco, San Francisco, CA USA; 5 Lawrence S. Bloomberg Faculty of Nursing, University of Toronto and St. Michael’s Hospital, Toronto, ON Canada; 6 Noncommunicable Diseases Theme, MRC/UVRI and LSHTM Uganda Research Unit, Entebbe, Uganda; 7 Institute for Policy Research, Northwestern University, Evanston, IL USA

**Keywords:** Scale development, Exclusive breastfeeding; human milk, Self-efficacy, Reliability, Validity, Psychometric evaluation

## Abstract

**Background:**

Increasing the prevalence of optimal breastfeeding practices, including exclusive breastfeeding for 6 months, could prevent an estimated 823,000 child deaths annually. Self-efficacy is an important determinant of breastfeeding behaviors. However, existing measures do not specifically assess *exclusive* breastfeeding self-efficacy, but rather self-efficacy for *any* breastfeeding. Hence, we sought to adapt and validate an instrument to measure exclusive breastfeeding self-efficacy.

**Methods:**

We modified and added items from Dennis’ Breastfeeding Self-Efficacy Scale-Short Form (BSES-SF). It was then implemented in an observational cohort in Gulu, Uganda at 1 (*n* = 239) and 3 (*n* = 238) months postpartum (clinicaltrials.gov NCT02925429). We performed inter-item and adjusted item-test correlations, as well as exploratory factor analysis and parallel analysis at 1 month postpartum to remove redundant items and determine their latent factor structure. We further applied confirmatory factor analysis to test dimensionality of the scale at 3 months postpartum. We then assessed the reliability of the scale and conducted tests of predictive and discriminant validity. Known group comparisons were made by primiparous status and correct breastfeeding knowledge.

**Results:**

The modification of the original BSES-SF to target exclusive breastfeeding produced 19 items, which were reduced to 9 based on item correlations and factor loadings. Two dimensions of the adapted scale, the Breastfeeding Self-Efficacy Scale to Measure Exclusive Breastfeeding BSES-EBF emerged: Cognitive and Functional subscales, with alpha coefficients of 0.85 and 0.79 at 3 months postpartum. Predictive and discriminant validity and known group comparisons assessments supported its validity.

**Conclusions:**

This version of the Breastfeeding Self-Efficacy scale, the BSES-EBF Scale, is valid and reliable for measuring exclusive breastfeeding self-efficacy in northern Uganda, and ready for adaptation and validation for clinical and programmatic use elsewhere.

**Electronic supplementary material:**

The online version of this article (10.1186/s12884-019-2217-7) contains supplementary material, which is available to authorized users.

## Background

Exclusive breastfeeding, i.e. the provision of human milk only and no other foods or liquids except medicines, saves lives and provides optimal and complete nutrition to infants [1]. It increases the survival, health and development of all children and contributes to the health and economic capital of women and their families [2, 3]. The World Health Organization, Centers for Disease Control and Prevention and American Academy of Pediatrics recommend early initiation of breastfeeding (within one hour of delivery), *exclusive* breastfeeding for the first 6 months of life and continued breastfeeding for at least two years [4–7]. Scaling up these optimal breastfeeding practices can prevent an estimated 823,000 child deaths and 20,000 breast cancer deaths every year [6]. Despite the substantive benefits from optimal breastfeeding [8] and, consequently, the risks of non-exclusive breastfeeding, exclusive breastfeeding rates remain low. Only 40% of infants younger than 6 months worldwide are exclusively breastfed [2, 9].

Many determinants have been identified that facilitate or deter breastfeeding behavior. Indeed, breastfeeding determinants operate across multiple dimensions of the socioecological model from the individual (mother-infant dyad) [10, 11], to the family, community, healthcare system, and cultural belief systems [12–14].

At the individual level, affective characteristics, or the feelings that capture people’s ways of responding, are fundamental to behavior [15]. Self-efficacy is an affective characteristic that has been identified as one of the strongest predictors of a range of behaviors, including breastfeeding [12]. Defined as a belief in one’s ability to succeed in executing a specific behavior [16], self-efficacy is critical to initiation and continued breastfeeding behavior [17]. As such, the ability to meaningfully assess perinatal levels of self-efficacy is valuable for both predicting breastfeeding initiation and breastfeeding behavior over time [18].

There are several existing instruments to measure breastfeeding self-efficacy [11, 19–22]. Of these instruments, Dennis’ Breastfeeding Self-Efficacy-Short Form (BSES-SF), which was developed for measuring breastfeeding self-efficacy among Canadian mothers, has been used most widely [11]. However, none of the instruments, including the BSES-SF, specifically measure *exclusive* breastfeeding self-efficacy; they measure confidence in *any* breastfeeding. Yet the focus on exclusive breastfeeding self-efficacy is important, especially in contexts where breastfeeding often continues well into the second year of life. Women in these settings are likely to feel confident in their ability to initiate and practice continued breastfeeding. However, because other foods and liquids are typically introduced before 6 months [23], self-efficacy for *exclusive* breastfeeding is likely to be low.

To measure self-efficacy, Bandura recommends using a behavior-specific technique, which includes capturing self-efficacy in one’s ability to perform the behavior and the skills or tasks necessary to complete the behavior [16]. Dennis applied these recommendations to her breastfeeding self-efficacy theory [17] and subsequent BSES-SF [11]. Essentially, breastfeeding self-efficacy captures a range of cognitive components, including 1) whether a mother chooses to breastfeed exclusively, 2) her effort expended, 3) how she perseveres when challenges arise, and 4) if she will be self-critical or self-encouraging to support her breastfeeding behavior, and 5) how she manages exclusive breastfeeding behaviors [17]. The other component, which includes behavioral skills associated with breastfeeding, captures 1) confidence in the baby’s latch, 2) identifying breast health issues and, 3) learning behavioral skills to overcome perceived or real barriers (e.g., hand-expressing breast milk, asking family members to not supplement with other foods). Thus, high levels of exclusive breastfeeding self-efficacy are foundational to successful exclusive breastfeeding initiation and continuation.

Therefore, to account for the specific tasks involved with exclusive breastfeeding and the beliefs associated with it, we adapted Dennis’ BSES-SF for the Ugandan context of women exclusively breastfeeding to create a scale to specifically measure self-efficacy for *exclusive* breastfeeding [11]. Our objectives were to (1) adapt the BSES-SF to emphasize exclusive breastfeeding for use in a setting where continued, but not exclusive, breastfeeding is common; (2) conduct psychometric testing of this adapted scale using breastfeeding and health-related factors.

## Methods

### Study settings and data collection

For this study we used data collected between October 10, 2012 and January 19, 2015 from the parent studies: Prenatal Nutrition and Psychosocial Health Outcomes Study (PreNAPS, ClinicalTrials.gov # NCT02922829), and Postnatal Nutrition and Psychosocial Health Outcomes Study (PostNAPS, ClinicalTrials.gov # NCT02925429). Data were collected from the antenatal care clinic at the Gulu Regional Referral Hospital in Gulu, Uganda. The purpose of the parent study was to explore links between food insecurity, psychosocial health and nutrition during pregnancy and postpartum.

Study procedures have been described elsewhere [24–26]. Briefly, 403 pregnant women (between 10 and 26 weeks) who were living < 30 km of Gulu Regional Referral Hospital, had knowledge of their HIV status met eligibility criteria and were offered enrollment in the PreNAPS study, and followed monthly during pregnancy if they consented. All PreNAPS participants who delivered after 9 May 2013 and had a live singleton birth were invited to participate in PostNAPS; all women accepted the invitation (*n* = 246). Data on socio-demographic factors, exclusive breastfeeding, exclusive breastfeeding social support, depression, wealth, and other health-related outcomes were collected at 1 week, then 1, 3, 6, 9 and 12 months postpartum; however, only data from 1 and 3 months were used for the development and validation of the BSES-EBF Scale [24, 26] because information about mother’s breastfeeding self-efficacy was asked at these two time points only.

### Participant characteristics and validation items

Demographic data included information on household size, parity, age, gravidity, and educational level. Wealth was operationalized based on participants’ possession of twenty different household assets outlined in the socio-economic module of the 2009–10 Uganda National Panel Survey [26, 27] using principle component analysis, which was then tertiled. Health information included maternal HIV status (determined at the postnatal care clinic prior to enrollment into the PostNAPs study), maternal depression (assessed using the Center for Epidemiological Studies depression scale) [28] and maternal general social support (assessed through an adapted version of the Duke UNC functional social support questionnaire) [29]. Similar methods for the development and validation of this scale were used in our previously published paper on exclusive breastfeeding social support [30].

Breastfeeding characteristics included assessments at one month postpartum of: participants’ correct knowledge of exclusive breastfeeding [determined by two questions which asked how long a baby could thrive on breastmilk alone (6 months) and the best way to feed a baby for the first six months (human milk only)], exclusive breastfeeding social support (determined by a validated scale that assessed three dimensions of social support: instrumental, emotional and informational) [30], and how the infants were fed based on participants’ recall of infant feeding practices.

### Exclusive breastfeeding self-efficacy item generation and adaptation

We modified and adapted the breastfeeding self-efficacy scale-short form (BSES-SF) to develop items for our exclusive breastfeeding self-efficacy scale [11]. The BSES-SF consists of 14 items and was designed to measure breastfeeding self-efficacy among postpartum women in Canada and has subsequently been adapted and validated in many settings worldwide [31–36], however we are not aware of a published adaptation for anywhere in sub-Saharan Africa. We modified two items of the 14 items and added five items to ensure our adapted version reflected maternal confidence and commitment to EBF in the Ugandan context (Table S1). Item generation and adaptation was based on a review of EBF literature and local knowledge of exclusive breastfeeding practices. As part of assessing the content validity of items, four experts in the fields of nutrition, public health, breastfeeding, and medicine reviewed the adapted scale to determine if the items were appropriate or tangential indicators of exclusive breastfeeding self-efficacy and relevant to a low-resource setting. These same experts reviewed each of the items for clarity and intent and other modifications made.

The item generation and adaptation of the BSES-SF produced an initial set of 19 items (Additional file 1: Table S1). Experts who reviewed the items suggested retaining all 14 items from the BSES-SF, with slight modification to two items to make them more specific to exclusive breastfeeding. Five additional items were also recommended to reflect exclusive breastfeeding self-efficacy: (1) “I can always make enough breast milk to satisfy my baby’s hunger”, (2) “I can always make good quality breast milk that has everything that my baby needs to grow well and be healthy”, (3) “I can always make decisions about how my baby is being fed”, (4) “I can always exclusively breastfeed without my baby receiving even a drop of water or any other liquid”, and (5) “I can always stop someone from trying to feed my baby liquids or foods other than breast milk, including purchased baby foods (e.g. infant formula, milk, porridge, juice, tea) before 6 months of age.” The response categories from the BSES-SF were retained for the modified scale, i.e. (1) not at all confident, (2) not very confident, (3) sometimes confident, (4) confident, and (5) very confident. The scale items were then forward translated into Acholi and Langi, and backward into English before being used for data collection. Our scale resulted in 19 questions which were administered in the PostNAPS study.

### Data analysis

We used data from the 1 month (*n* = 239) and 3 month (*n* = 238) postpartum visits. Data were analyzed in six phases: descriptive analysis, item reduction and functionality, extraction of factors, test of dimensionality, reliability and validity. Data were analyzed using Mplus v. 8.0 (Los Angeles, CA: Muthén & Muthén) and STATA v.14 (College station, TX: StatCorp LP).

#### Descriptive analysis

Descriptive statistics including proportions, means and standard deviations of the demographic, health, and breastfeeding behavior variables were first estimated.

#### Item reduction and functionality

Following similar methodological techniques to the development of our exclusive breastfeeding social support scale [30], we then conducted item reduction tests that included items that were functional, parsimonious, and internally consistent [37]. For the initial 19 items, we examined response proportions, means, and variances between items for adequate variance. Items were dropped if they were poorly functional, i.e. they had an item-total correlation of less than 0.30. Secondly, items with 5 or more inter-item correlations below 0.2 were dropped to increase homogeneity [38]. Since all 19 items were measured at the ordinal level and consisted of 5 categories, we estimated polychoric (inter-item) and polyserial (item-total) correlations using both maximum likelihood and weighted least squares with mean and variance adjustment estimators to determine which items should be dropped from the tentative scale [38–40].

We then re-assessed the inter-item and adjusted item-total correlations on the remaining items to ensure our items were functional. We hypothesized that this set of items would correlate highly with the underlying construct we intended them to measure [41].

#### Extraction of factors

Extraction of factors serves two purposes in scale development; 1) the number of factors that fit a set of items is determined and, 2) it contributes to construct validity. The emphasis is on the number of factors, the salience of factor loading estimates, and the relative magnitude of residual variances [39, 42]. We used two techniques to identify the appropriate number of factors to retain at 1 month postpartum. Exploratory factor analysis (EFA) was used with Kaiser eigenvalue > 1 rule and Cattell’s scree test to determine the number of factors to retain [43–45]. At this stage, item loadings < 0.30 were dropped because they fell below the threshold and were considered misrepresentative of the factor [40, 46]. We operationalized items as continuous and therefore used oblique rotation with maximum likelihood (ML) for the extraction process [47]. To validate the factors retained, we did a sensitivity test applying Horn’s parallel analysis [48]. Meaningful model fitness was determined by using a number of model fit indices with satisfactory thresholds including, chi-square test of model fit, Akaike Information Criterion (AIC), Bayesian Information Criterion (BIC), Root Mean Square of Error Approximation (RMSEA≤0.10), Tucker Lewis Index (TLI ≥ 0.95), Comparative Fit Index (CFI ≥ 0.95) and Standardized Root Mean Square Residual (SRMR≤0.08) [49–54]. The results from this analysis provided the hypothesized factor structure to be tested with data at a later time point (i.e. from the 3-month postpartum visit).

#### Test of dimensionality

The test of dimensionality is a confirmatory test where the hypothesized factors or factor structure extracted from a previous model is tested [40, 55]. Our hypothesized factor structure was tested using confirmatory factor analysis (CFA) using oblique rotation with maximum likelihood estimator. A number of model fit indices with satisfactory thresholds were used to determine meaningful model fitness for the test of dimensionality. This included chi-square test of model fit, AIC, BIC, RMSEA, TLI, CFI, and Weighted Root Mean Square Residual [49–54].

#### Model modification

Model modification is used in confirmatory factor analysis to improve model fitness using modification indices while providing remedies for discrepancies between proposed and estimated models [53, 56]. Modifications can be made to items if they are theoretically justifiable, few in number, and they do not have a major impact on estimates of other parameters in the model [53, 56, 57]. We did this in Mplus and then examined the results for the largest modification indices and for error terms associated with the observed indicators. Partial correlations were then estimated on identified error terms to improve model fitness [53, 56]. With a stronger identified model, we tested for the reliability and validity of the scale.

#### Reliability of exclusive breastfeeding self-efficacy scale

Reliability is the degree of consistency when a scale or measure is repeated under identical conditions [58]. We assessed the reliability of the scale using Cronbach’s coefficient alpha and coefficient of stability [38, 59]. The coefficient alpha assesses the internal consistency of the scale i.e. the degree to which the set of items in the scale co-vary, relative to their sum score [38, 42, 60]. Reliability was assessed for EBFSE Scale at 1 and 3 months postpartum using Cronbach’s alpha. An alpha coefficient of 0.70 was determined as acceptable threshold for reliability; 0.80 and above is preferred for the psychometric quality of scales [46, 59, 61].

The coefficient of stability is used to assess how consistent a participant’s performance on a scale is when repeated over time. We assessed coefficient of stability through test-retest reliability [38]. This was indexed by a correlation coefficient at 1 and 3 months postpartum. The acceptable threshold for a test-retest analysis is a correlation of 0.70 for 100 participants across a three month interval [53].

#### Validity of exclusive breastfeeding self-efficacy scale

Validity of a scale is how well it measures what it is intending to measure [38]. The final scale items and associated dimensions were tested for their predictive and discriminant validity, as well as known group comparison using data at 1 month postpartum (*n* = 239).

Predictive validity is how well test scores predict outcomes (i.e., exclusive breastfeeding) in the future. We assess predictive validity using a Student’s *t-*test followed by logistic regression [38, 60]. Predictive validity of the two-dimensional BSES-EBF Scale at one and three months was assessed against exclusive breastfeeding behavior at one, three and six months postpartum. We hypothesized that participants who reported breastfeeding would have higher scores on the BSES-EBF Scale and both dimensions of the scale would predict exclusive breastfeeding behavior.

Discriminant validity is the degree to which a scale is able to capture a distinct construct and not reflective another construct(s) [38]. We assessed discriminant validity through a two-factor latent variable model (LVM), with a factor for each of the two constructs measured by first, the two dimensions of BSES-EBF Scale and second, Exclusive Breastfeeding Social Support. The next test was conducted between BSES-EBF and maternal depression, as the two variables have been found to be divergent [11]. We then calculated the point and interval estimate of the interrelationships giving us the ‘true’ correlational estimates between the unobservable constructs for the population [38]. Discriminant validity was assessed by predictably low/weak correlations between BSES-EBF Scale score and the other constructs not measuring BSES-EBF [38, 62].

We also assessed known group comparisons, i.e. groups we expected to experience higher scores on BSES-EBF Scale. This is an approach that examines the distribution of a newly developed scale score over known binary items [38, 62]. We used breastfeeding knowledge and primiparous status as the groups and hypothesized that participants with correct breastfeeding knowledge and multiparae would have significantly higher BSES-EBF Scale mean scores than participants with incorrect breastfeeding knowledge and primiparae.

Convergent validity was not assessed as there was no construct similar to exclusive breastfeeding self-efficacy in our data. Additionally, criterion concurrent validity was not assessed, as no ‘gold standard’ has been developed for measuring exclusive breastfeeding self-efficacy. Nonetheless, the use of these three approaches provide multiple evidence to support the validity of the BSES-EBF Scale [38].

## Results

### Participant characteristics

The mean age of participants was 25.2 with a range of 16 to 42 years (Table 1). Each woman had a mean of 1.6 children with an average household size of 4.6. One-quarter (23.1%) were primiparous and 36.8% were HIV positive. As for education, approximately half (55.7%) had less than primary education.

**Table 1:** Descriptive statistics of socio-demographic and breastfeeding characteristics of Ugandan study participants at 1 and 3 months postpartum (*n* = 239)

Socio-demographic characteristics (range or %)	N/ (%) | Mean (SD)
Maternal age (16–42)	25.2 (5.3)
Number of children (0–8)	1.6 (1.5)
Household size (1–13)	4.6 (2.2)
Primiparous (%)	55 (23.0)
HIV positive status (%)	88 (36.8)
Gravidity (1–10)	2.9 (1.8)
Maternal Education (%)	
Less than Primary	131 (55.7)
Wealth (%)	
Poorer	82 (34.1)
Middle	80 (33.5)
General Social Support (GSS) (10–30)	19.1 (4.2)
CESD^1^-Depression scale (0–53)	18.5 (10.9)
Breastfeeding characteristics at 1 month postpartum	
Correct breastfeeding knowledge^2^ (%)	139 (58.2)
Exclusive Breastfeeding (EBF) (%)	151 (63.9)
Instrumental EBFSS^3^ (3–9)	4.8 (1.7)
Emotional EBFSS (8–24)	13.2 (3.7)
Informational EBFSS (5–15)	7.4 (2.3)
BSES-EBF Scores	
Cognitive subscale^4^ (1 month) (4–20)	13.5 (4.1)
Functional subscale (1 month) (5–25)	17.2 (4.3)
Cognitive subscale (3 month) (4–20)	13.6 (4.1)
Functional subscale (3 months) (5–25)	17.7 (4.3)

Notes: ^1^*CESD* Center for Epidemiological Studies-Depression Scale, *N* Sample size, *SD* Standard Deviation; ^2^women were considered as having correct breastfeeding knowledge if they answered both knowledge questions correctly; ^3^EBFSS = Exclusive breastfeeding social support; ^4^Breastfeeding Self-Efficacy Scale to Measure Exclusive Breastfeeding

The General Social Support scale produced a mean score of 19.1 ± 4.2, with a Cronbach’s alpha of 0.86. Mean CES-D score was 18.5, with a Cronbach’s alpha of 0.89 (Table 1).

Of the 239 participants, 58.2% were considered as having adequate exclusive breastfeeding knowledge. At one month postpartum, 63.9% reported exclusively breastfeeding their infants (Table 1). We assessed breastfeeding social support using the Exclusive Breastfeeding Social Support scale which consists of three breastfeeding dimensions: Instrumental, Informational and Emotional [30]. At one month postpartum, the instrumental exclusive breastfeeding social support score was 4.8, with a Cronbach’s alpha of 0.78; the emotional exclusive breastfeeding social support score was 13.2 with a Cronbach’s alpha of 0.85; and the informational exclusive breastfeeding social support score was 7.4 with a Cronbach’s alpha of 0.83.

### Item reduction and functionality

Our criteria for item reduction and functionality resulted in the deletion of 8 of the 19 items. All eight items had less than 0.2 inter-item correlations with at least 5 of the 19 items. Five out of the 8 items deleted had item-total correlations and adjusted item-total correlations of < 0.3 (Additional file 1: Table S1). A re-estimation of the 11 remaining items produced inter-item correlation coefficients ranging from 0.11 to 0.86 (Table 2). The mean for item-total correlations for all 11 items was 0.63, with a range of 0.40 to 0.79. The mean for adjusted item-total correlations was 0.52, with a range of 0.28 to 0.72 (Table 2).

**Table 2:** Descriptive statistics and item correlation coefficients for Exclusive Breastfeeding Self-Efficacy Scale among postpartum women in northern Uganda (n = 239)

Variable	Response Percentages							Correlation Matrix										
	Mean	Variance	1	2	3	4	5	1	2	3	4	5	6	7	8	9	10	11
Enough Milk	3.65	1.78	12.55	6.69	17.15	30.13	33.47	1.00										
Challenging Tasks	3.16	1.20	8.37	19.67	27.62	35.98	8.37	0.32	1.00									
EBF Milk	3.31	1.76	16.74	9.21	17.57	38.91	17.57	0.30	0.36	1.00								
Manage BF	3.72	1.10	5.44	7.95	15.90	50.12	20.50	0.13	0.24	0.19	1.00							
Continue EBF	3.40	1.29	10.04	8.79	25.94	41.84	13.39	0.11	0.31	0.53	0.33	1.00						
Satisfy BF	3.22	1.53	10.88	22.59	12.97	41.00	12.55	0.43	0.22	0.29	0.13	0.16	1.00					
Deal BF	3.73	1.51	7.95	9.62	16.32	33.89	32.22	0.38	0.23	0.29	0.17	0.11	0.47	1.00				
BF Every feeding	3.09	1.05	10.88	12.13	37.66	35.56	3.77	0.34	0.31	0.51	0.19	0.41	0.35	0.31	1.00			
Keep BF demands	3.47	1.33	10.04	8.79	20.08	46.03	15.06	0.43	0.34	0.51	0.23	0.38	0.55	0.46	0.86	1.00		
EBF Liquid	3.23	1.84	19.25	7.95	20.92	34.31	17.57	0.26	0.29	0.80	0.17	0.52	0.30	0.26	0.49	0.51	1.00	
Stop Other foods	3.55	1.51	10.46	8.37	20.08	37.66	23.43	0.22	0.26	0.53	0.19	0.36	0.22	0.27	0.36	0.30	0.66	1.00
Item-total correlation								0.56	0.52	0.79	0.40	0.60	0.59	0.56	0.72	0.79	0.78	0.63
Adjusted item-total correlation								0.43	0.41	0.70	0.28	0.49	0.47	0.43	0.65	0.72	0.69	0.52

Notes: Response categories for each variable included 1 = Not at all confident, 2 = Not very confident, 3 = Sometimes confident, 4 = Confident, 5 = Very confident; Median for all items was 4.0 except ‘challenging task’ and ‘BF Every feeding’, which was 3.0; All results obtained from women at one month postpartum

**Fig. 1:**
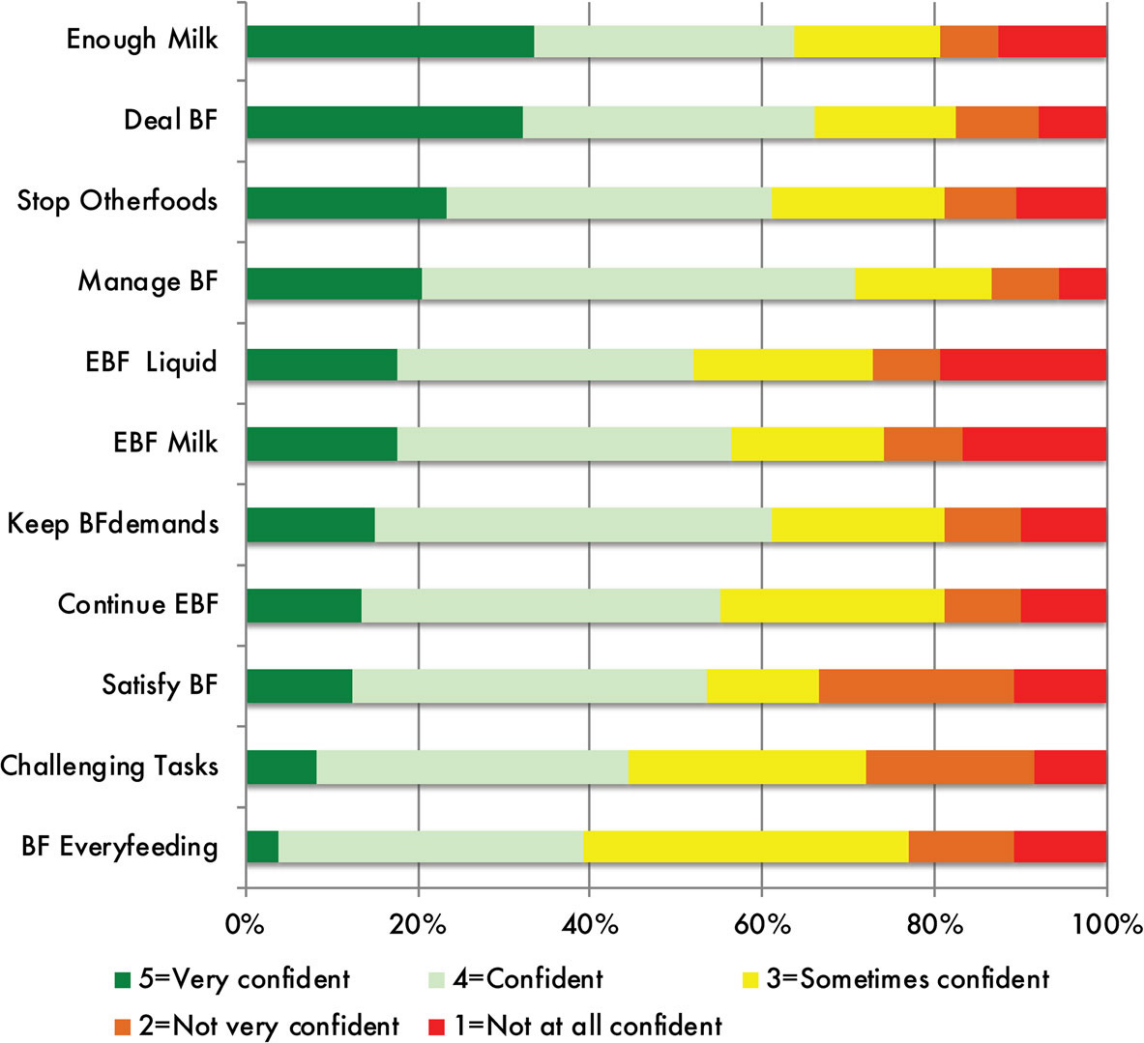
Item response clustered bar graph showing response categories for 11 Breastfeeding Self-Efficacy Scale to Measure Exclusive Breastfeeding (BSES-EBF) items (*n* = 239). “Manage BF” and “Challenging Tasks” were ultimately dropped after exploratory factor analysis; All items are examined at one month postpartum

The median for all 11 items was 4.0, except for “challenging task” and “BF Every feeding”, and a mean range of 3.09 to 3.73 (Fig. 1**,** Table 2). Items indicating greater confidence included “Enough milk”, “Deal BF”, and “Stop Other foods”. Items where respondents indicated lower confidence included “EBF Liquid” and “EBF milk” (Fig. 1**,** Table 2).

### Extraction of factors

**Fig. 2:**
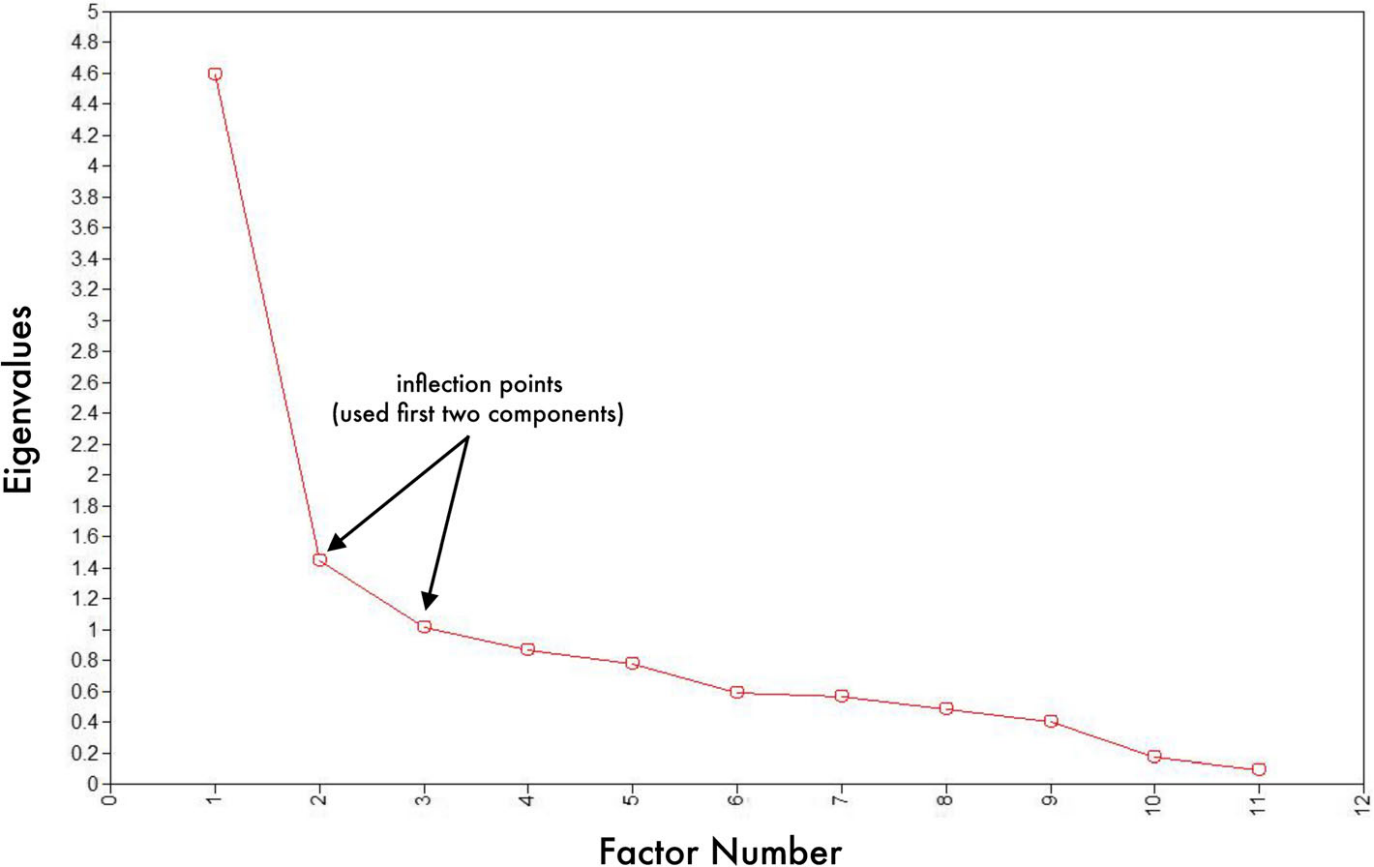
Scree plot showing retained scale factors using exploratory factor analysis at one month postpartum

Two factors were identified from the 11 items using ML estimator (Table 3). Three eigenvalues > 1 were produced off the Geomin oblique rotation (Table 4) and accounted for 60.9% of the variance in the data. Although three eigenvalues were produced, only two factors were tenable statistically. An examination of the scree plot showed a steep curve that leveled off at factor number 2 with a corresponding eigenvalue > 1 (Fig. 2). This also pointed to a two-dimensional scale. The sensitivity test using Horn’s parallel analysis also produced a two-factor model (Additional file 2: Figure S1).

**Table 3:** Exploratory factor loading results of 11 items indicative of Exclusive Breastfeeding Self-Efficacy Scale among postpartum women in northern Uganda

	One-factor model	Two-factor model	
	1	1	2
Enough Milk	0.40		**0.30**
Challenging Tasks	0.39	0.24	
EBF Milk	0.74	**0.85**	
Manage BF	0.30		
Continue EBF	0.54	**0.51**	
Satisfy BF	0.47		**0.47**
Deal BF	0.41		**0.36**
BF Every feeding	0.78		**0.74**
Keep BF demands	0.80		**1.02**
EBF Liquid	0.74	**0.90**	
Stop Other foods	0.53	**0.68**	

Notes: All items in exploratory factor analysis will have loadings on all factors; shown here are only the largest factor loadings (cut-off = 0.3) that were significant (*p* ≤ 0.05). ‘Manage BF’ was removed from the final model because it had no significant factor loading in the Two-factor model; ‘Challenging task’ was dropped because it was below the threshold of 0.3

**Table 4:** Model Fit Indices of Factor Extraction at 1 month postpartum and test of dimensionality at 3 months postpartum

Factor Extraction (*n* = 239)									
Rotation	Analytical Technique	AIC^1^	BIC^2^	χ2^3^	*df* ^*4*^	RMSEA^5^	CFI^6^	TLI^7^	SRMR^8^
Geomin Oblique Rotation	**EFA** ^**9**^ **(11 items)**								
Eigenvalues	**4.29, 1.40, 1.01**								
	**One-factor model**	7747.46	7862.18	344.64	44	0.17	0.70	0.62	0.08
	**Two-factor model**	**7521.89**	**7671.38**	99.07	34	**0.09**	**0.93**	**0.89**	**0.06**
**Test of Dimensionality (*n*** **= 238)**		**AIC**	**BIC**	**χ2**	***df***	**RMSEA**	**CFI**	**TLI**	**WRMR** ^10^
Geomin Oblique Rotation (Initial CFA test)	**CFA** ^11^ **(2 factors)**	**5837.01**	**5934.11**	90.62	26	**0.10**	**0.94**	**0.91**	**0.61**
**After re-specification** (Geomin Oblique Rotation)	**CFA (2 factors)**	**5808.44**	**5912.48**	58.05	24	**0.08**	**0.97**	**0.95**	**0.60**

Notes: ^1^**AIC** = Akaike, ^2^**BIC** = Bayesian, ^3^**χ2** = chi-square goodness of fit statistic; ^4^***df*** = degrees of freedom; ^5^**RMSEA (≤0.08)** = Root Mean Square Error of Approximation; ^6^**CFI (≥0.95)** = Comparative Fit Index; ^7^**TLI (≥0.95)** = Tucker Lewis Index; ^8^**SRMR (≤0.08)** = Standardized Square Root Mean Residual; ^9^**EFA** = Exploratory Factor Analysis; ^10^**WRMR** (≤1.0) = Weighted Root Mean Square Residual; ^11^CFA = Confirmatory Factor Analysis; All goodness-of-fit tests were statistically significant at p < 0.0001

With the re-examination of factor loadings, one item – ‘Challenging task’ – with a lower factor loading (< 0.30) and a second item ‘Manage BF’ with a lower (< 0.30) and a non-significant factor loading were dropped from the list of items in the two-factor model (Table 3). In all, a total of 10 items were dropped, seven from the original items adapted and three from the newly added items.

The remaining 9 items could be grouped into what we identified to be the Cognitive and Functional BSES-EBF subscales. The cognitive subscale consisted of four items reflecting the belief of participants to exclusively breastfeed (e.g. Stop Other foods). The functional subscale consisted of 5 items reflecting the competence, and ability of the participants to breastfeed (e.g. Enough Milk). Each factor had a Cronbach’s alpha value greater than 0.70. Results from the analysis indicated satisfactory model fitness: χ2 (34) = 99.07, *p* < 0.001; RMSEA (0.09), CFI (0.93), SRMR (0.06), AIC (7521.89), and BIC (7671.38) (Two factor model**,** Table 4).

### Test of dimensionality

Based on the EFA results, we hypothesized that the 9 items will represent a two-dimensional scale. Using CFA, we tested the hypothesis with data from 3 months postpartum (*n* = 238). The overall fit of the model for the initial test was poor on an absolute basis: χ2 (26) =90.62, *p* < 0.001; however, the descriptive model fit statistics indicated satisfactory model fitness: RMSEA (0.10), CFI (0.94), SRMR (0.06), AIC (5837.01), and BIC (5934.11) (Table 4).

### Model modification

To improve model fitness, we reran the analysis requesting for “modification indices” (MI) in Mplus using an MI of 3.84 to identify items that needed re-specification [53, 56]. A recommended change based on an MI greater than 3.84 will reduce χ2 by a statistically significant amount [57]. The modification results suggested two WITH statements (partial correlations), which referred to covariance between error terms associated with 2 identified paired items (Additional file 3: Table S2). By correlating the error terms associated with D2L10 and D2M10, we would reduce χ2 by 19.49 points; the covariance estimate would change to 0.33 (Additional file 3: Table S2). Also, by correlating the error terms associated with D2S10 WITH D2R10, we would reduce χ2 by 13.94 points and the covariance estimate would change to 0.33. These estimated results made it statistically justifiable to correlate the two pairs of items for model improvement.

After re-specification of the model, our model fit indices improved greatly, without having a major effect on the estimates of other parameters in the model (Table 4).

**Fig. 3:**
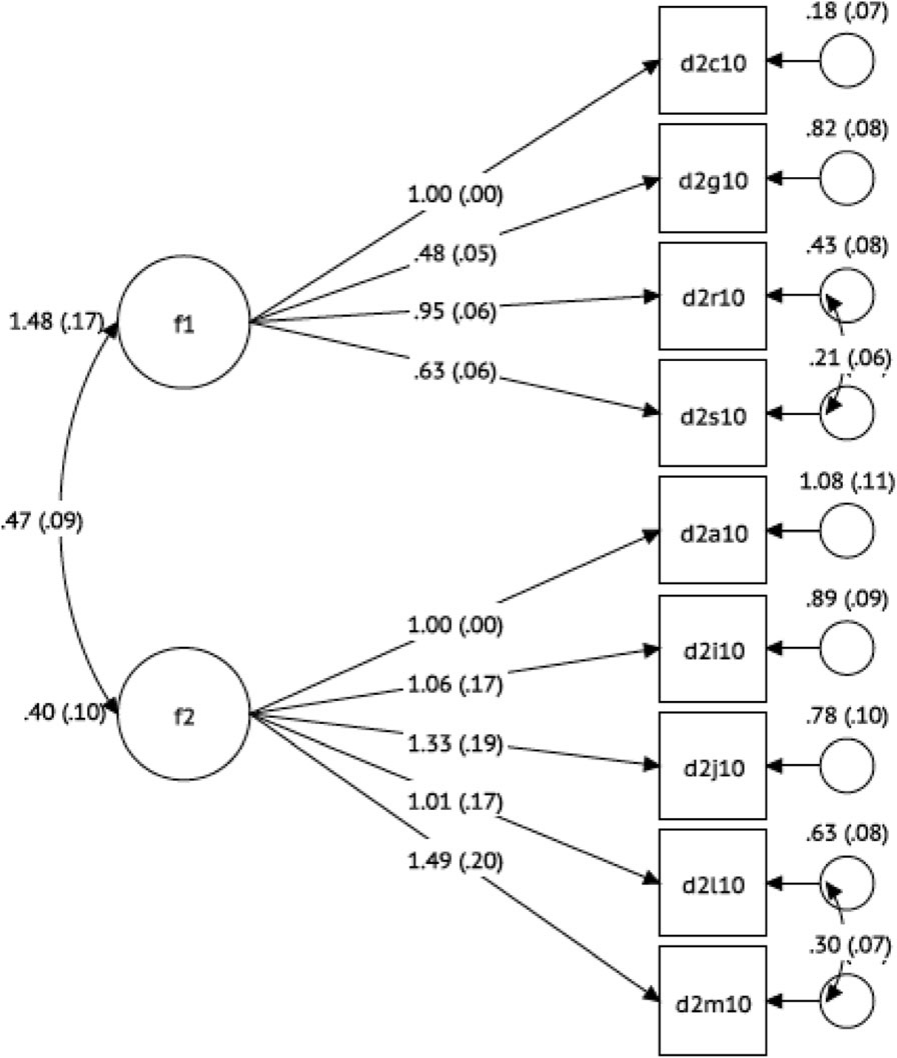
CFA model estimates with residual errors showing a bi-dimensionality of the Breastfeeding Self-Efficacy Scale to Measure Exclusive Breastfeeding (BSES-EBF) Scale at 3 months postpartum (*n* = 238)

All model fit indices provided evidence for a two-dimensional scale: χ2 (24) =58.05, *p* < 0.001; RMSEA (0.08), CFI (0.97), TLI (0.95), WRMR (0.95), AIC (5837.01), and BIC (5912.48). Based on the results, a two-dimensional scale was accepted as an appropriate fit for our data giving a finalized scale of 9 questions reflecting exclusive breastfeeding self-efficacy (Fig. 3**,** Table 5).

**Table 5:** Final Exclusive Breastfeeding Self-Efficacy Scale items validated for use among women in northern Uganda

Tell me how you would rank your confidence on a scale of 1 to 5 for each statement:		
Domain	Item Label	Items
Cognitive	EBF Milk	I can always give my baby only breast milk without using animal milk, formula, or other liquids or foods as a supplement
	Continue EBF	I can continue exclusively breastfeeding for as long as I want
	EBF Liquid	I can always exclusively breastfeed without my baby receiving even a drop of water or any other liquid
	Stop Other Foods	I can always stop someone from trying to feed my baby liquids or foods other than breast milk, including purchased baby foods (e.g. infant formula, milk, porridge, juice, tea [whatever is commonly given]), before 6 months of age
Functional	Enough Milk	… determine that my baby is getting enough milk*
	Satisfy BF	… be satisfied with my breastfeeding experience*
	Deal BF	… deal with the fact that breastfeeding can be time consuming*
	BF Every Feeding	… continue to breastfeed my baby for every feeding*
	Keep Bf Demands	… manage to keep up with my baby’s breastfeeding demands*

Notes: Rating scale: 1 = Not at all confident; 2 = Not very confident; 3 = sometimes confident; 4 = confident; 5 = very confident; Functional items reflect items unchanged from the BSES-SF and Cognitive items are new/modified items

*For complete phrasing, please contact Dr. Cindy Lee Dennis: cindylee.dennis@utoronto.ca

In order to obtain the composite score for each of the two dimensions, we calculated the sum of responses for scale items at one and three months postpartum. At one month, the Cognitive subscale had a mean of 13.5 ± 4.1 and a range of 4–20 and the Functional subscale had a mean of 17.2 ± 4.3 and range of 5–25. The range for each of the sub-scales remained the same at 3 months postpartum; however, there were slight increases in each of the scores (Table 1).

### Reliability of the BSES-EBF scale

Reliability for the two subscales was measured using Cronbach’s alpha and test-retest correlation at 1 and 3 months postpartum. The reliability test for the two subscales, Cognitive and Functional, produced respective Cronbach’s alpha of 0.82 and 0.77 at month 1, and 0.85 and 0.79 at month 3 (Table 6). These values were consistently above the published satisfactory (0.70) thresholds for scale reliability [59, 61]. All corrected item-total correlations were positive and ranged between 0.28 and 70.

**Table 6:** Reliability scores for the Cognitive and Functional sub-scales of BSES-EBF Scale at 1 and 3 months postpartum

EBFSS Subscales	Average Inter-item covariance (1 month)	Average Inter-item covariance (3 months)	Cronbach’s Alpha Coefficient (1 month)	Cronbach’s Alpha Coefficient (3 months)	Coefficient of Stability
Cognitive (4 items)	0.8598	0.894	0.8219	0.8523	0.54 (95% CI: 0.44, 0.62; *p* < 0.001)
Functional (5 items)	0.5758	0.5743	0.7677	0.7903	0.22 (95% CI: 0.09, 0.34; *p* < 0.001)

We then assessed test-retest reliability by correlating the scores of each subscale at month 1 and month 3 to produce the coefficient of stability. Our estimation produced a significant correlation coefficient of 0.54 for the Cognitive subscale, and 0.22 for the Functional subscale. Both coefficients were below the threshold of 0.70 (Table 6).

### Validity of BSES-EBF scale

#### Predictive validity

For predictive validity, we regressed exclusive breastfeeding behavior simultaneously on each of the subscales at 1, 3 and 6 months postpartum (Additional file 4: Table S3). Initial t-test results at one-month postpartum showed participants who exclusively breastfeed consistently had higher scores on both Cognitive (10.77 vs. 13.3, *t* = − 2.98, *p* = 0.003) and Functional (14.00 vs. 17.35, *t* = − 2.46, *p* = 0.014) subscales. Our logistic regression model showed that the Cognitive sub-scale at 1-month postpartum was predictive of EBF at 1 month and 3 months, but not at 6 months postpartum (Additional file 4: Table S3). The Functional sub-scale at 1-month post-partum was predictive of EBF behavior at 1 month postpartum. The OR was in the expected direction, but was not statistically significant. Furthermore, the Cognitive sub-scale at 3 months was predictive of EBF at 3 months (OR = 1.15, 95% CI:1.08, 1.21; *p* = 0.000) and 6 months (OR = 1.19, 95%CI: 1.09, 1.29; *p* < 0.001) postpartum. The Functional subscale at 3 months postpartum was predictive of EBF behavior at 3 months (OR = 1.07, 95%CI:1.03, 1.12; *p* = 0.001), but not the relationship was not statistically significant at 6 months. BSES-EBF scores predicted exclusive breastfeeding both concurrently and three months later.

#### Discriminant validity

**Fig. 4:**
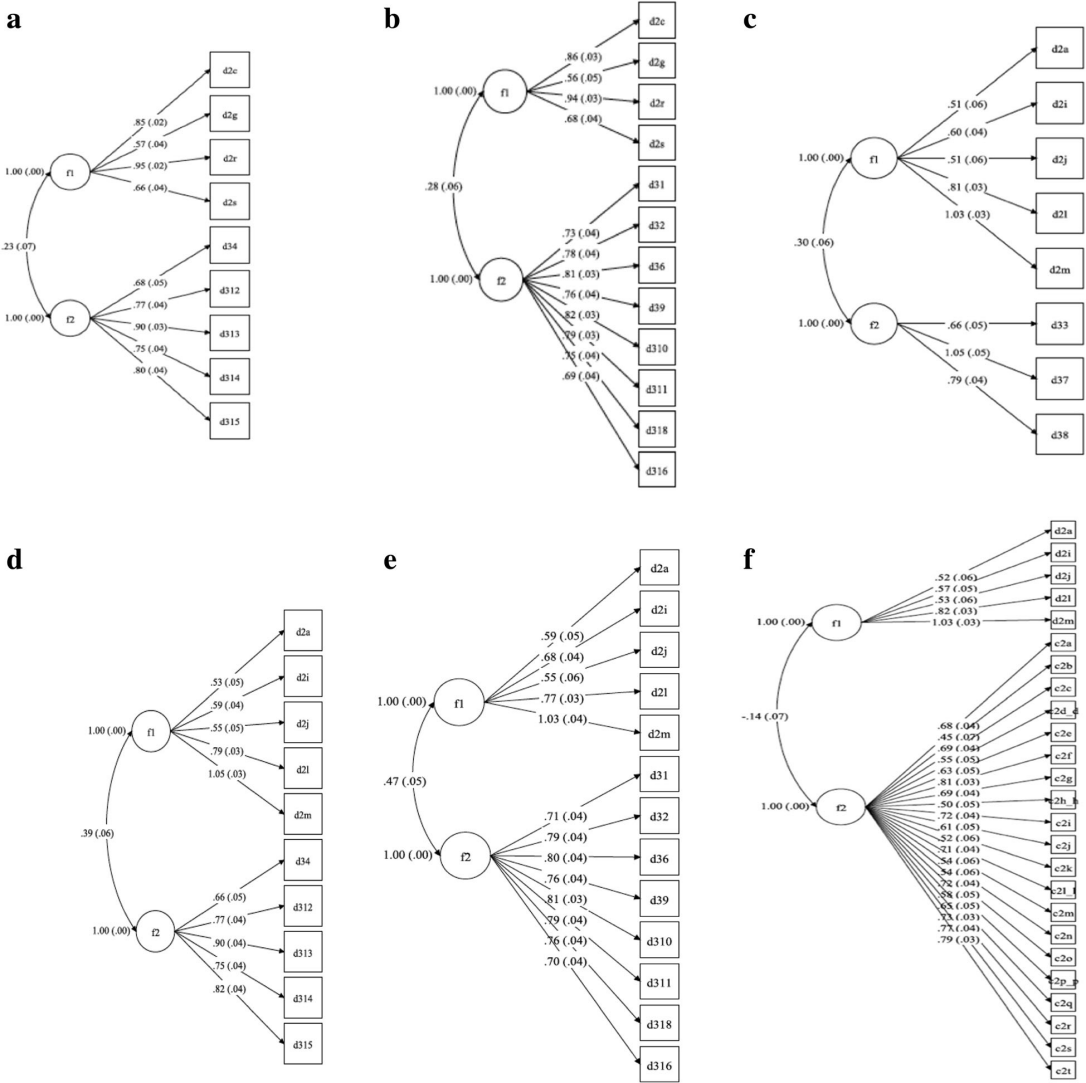
Latent variable model standardized estimates and population correlation coefficient for the interrelationship between **a.** the Cognitive subscale of the BSES-EBF and Informational EBFSS; **b.** the Cognitive subscale of the BSES-EBF and Emotional EBFSS; **c.** the Functional subscale of the BSES-EBF and Instrumental EBFSS; **d** the Latent variable model standardized estimates and population correlation coefficient for the interrelationship between Functional subscale of the BSES-EBF and Informational EBFSS; **e.** the Latent variable model standardized estimates and population correlation coefficient for the interrelationship between Cognitive subscale of the BSES-EBF and Emotional EBFSS; **f.** the Functional subscale of the BSES-EBF and CESD Score.

We assessed discriminant validity using a two-factor latent variable model (LVM) of the BSES-EBF Scale score and two constructs, Exclusive Breastfeeding Social Support (EBFSS) and maternal depression. The model fit indexes suggested the LVMs were tenable for 6 out of the 8 interrelationships (Additional file 5: Table S4). The population correlation estimates between the Cognitive subscale and Informational EBFSS (ρ = 0.23, 95% CI: 0.10, 0.36; *p* = 0.001) and Emotional EBFSS (ρ = 0.28, 95% CI: 0.16, 0.40; *p* = 0.001) showed strong evidence of a weak (linear) relationship between exclusive breastfeeding self-efficacy and EBFSS (Fig. 4a & 4b). Similarly, a weak correlation was evident between the Functional subscale and Instrumental EBFSS (ρ = 0.31, 95% CI: 0.18, 0.43; *p* = 0.001), Informational EBFSS (ρ = 0.39, 95% CI: 0.28, 0.50; *p* = 0.001), Emotional EBFSS (ρ = 0.47, 95% CI: 0.38, 0.57; *p* = 0.001), and depression (ρ = − 0.14, 95% CI: -0.28, − 0.01; *p* = 0.05) **(**Fig. 4**-**4f). In both constructs, the correlation estimates ranged from − 0.14 to 0.57.

#### Known group comparisons

In the final step, known group comparisons, we compared the position of multiparous vs. primiparous participants and participants with correct breastfeeding knowledge vs. incorrect breastfeeding knowledge on the two subscales (Additional file 4: Table S3). There were no significant differences between participants who were multiparous and primiparous, although multiparous participants did have slightly higher mean scores. However, participants with correct breastfeeding knowledge had higher mean scores on Cognitive (14.42 vs.12.24, *t* = − 4.19, *p* = 0.001) and Functional (17.41 vs.16.81, *t* = − 1.07, *p* = 0.28) subscales than participants without correct breastfeeding knowledge.

## Discussion

We adapted the BSES-SF scale to measure exclusive breastfeeding self-efficacy among Ugandan women. The result was a final 9-item BSES-EBF Scale with two dimensions, Cognitive and Functional, that showed strong validity and reliability in measuring exclusive breastfeeding self-efficacy in northern Uganda. Its utility is in its specificity to *exclusive* breastfeeding self-efficacy over existing self-efficacy scales, including the original BSES-SF upon which this was based, that assess confidence in *any* breastfeeding, and its application for low-resource settings. It will be of particular use in any setting where breastfeeding is a widely practiced cultural norm but exclusive breastfeeding is not common.

The BSES-EBF Scale was reliable at 1 and 3 months postpartum (Table 6). The Cronbach’s alpha for the two sub-scales were all above satisfactory thresholds with the Functional subscale above 0.70 and 0.80 for the Cognitive subscale [46, 59, 61]. However, the magnitude of the alpha coefficients of the subscales (0.82 and 0.77) was lower than Dennis’ or other scales [19–21]. This is partially due to our subscales having fewer items, which produce lower Cronbach’s alpha [38]. However, even these slightly lower Cronbach’s alpha estimates, together with the other demonstrations of reliability – adjusted item-total correlations and the test-retest reliability – support our scale as having the requisite psychometric reliability. The lower coefficient of stability indicates the need to test for reliability across time and different populations.

Our modified scale, the BSES-EBF Scale, is distinct from Dennis’ BSES-SF and her longer Breastfeeding Self-Efficacy Scale, in several ways. First, the BSES-EBF Scale is shorter (9 items), compared to the BSES-SF (14 items) or the original BSES scale (33 items) [11, 63]. The brevity of the BSES-EBF Scale is a significant advantage, in that it requires fewer resources, including time, to administer. Second, the BSES-EBF Scale targets the assessment of exclusive breastfeeding, rather than any breastfeeding or mixed-feeding.

Validity was tested using construct validity with both exploratory and confirmatory factor analysis, predictive validity, discriminant validity (correlational analysis), and known group comparisons. Previous scales developed on this subject used at most 3 out of the 4 validation procedures, except Dennis [10], who used all four. Specifically, Cleveland and McCrone [19], Hill and Humenick [20], and Wells et al. [64] assessed construct validity, predictive validity, and correlations, without any assessment of known group comparisons. Wells et al. [21] measured construct validity and known group comparison, with no measurement of predictive validity. Our approach to discriminant validity differs significantly from all existing scales that used correlational analysis, as it uses latent variable modeling to discriminate between exclusive breastfeeding self-efficacy and other constructs within the population [38, 65]. Thus, we are able to conclude with a high degree of confidence that our scale can discriminate from other constructs such as exclusive breastfeeding social support and maternal depression. By applying a rigorous and multi-step approach, these validation techniques suggest support for the validity of our scale analogous to most existing breastfeeding self-efficacy scales.

The results of this study have programmatic and policy implication for supporting exclusive breastfeeding in northern Uganda. First, our descriptive results of BSES-EBF items showed high confidence with items depicting behavioral skills and lower confidence in items describing beliefs and attitudes towards EBF (Fig. 1**,** Table 2). Participants had lower confidence in their ability to give their infants only breast milk without using animal milk, formula or other liquids or foods as a supplement. They also had a much lower confidence about their ability to not give infants any water. Based on the findings of Cleveland and McCrone [19], which show personal efficacy beliefs to be a significant predictor of exclusive breastfeeding, the use of educational interventions to change participants’ beliefs about their ability to exclusively breastfeed has the potential to significantly increase exclusive breastfeeding among women in northern Uganda.

A second programmatic implication may be that the use of the BSES-EBF scale by health practitioners in clinics and the community may be able to target those with lowest perceived exclusive breastfeeding self-efficacy, i.e. those most in need of support. Interventions aimed at following-up with mothers who score low on the BSES-EBF Scale could significantly improve their breastfeeding behaviors through early intervention. Third, the two domains: functional and cognitive, of the BSES-EBF Scale can differentiate *where* mothers may be succeeding or struggling with exclusive breastfeeding practice, which can help target appropriate support. Finally, this scale may be useful programmatically in that it can be administered to participants during the evaluation of programs and policy initiatives aimed at increasing commitment to exclusive breastfeeding.

Although there are many strengths, there are several limitations in our study. First, the correlational effect we find between the different sub-scales at month one and three may be due to common method variance. Hence, it will be appropriate to test the hypothetical factor structure on a new sample to ascertain if they have the same meanings, latent factors, and factor loadings [38, 60]. Second, the lower coefficients of stability in the test-retest reliability may be due to behavioral changes which occur with lactating women after breastfeeding for some time [66, 67]. However, these lower coefficients reinforce the need to retest this scale in a new population.

Indeed, there is plenty of opportunity for further work on the BSES-EBF Scale. First, estimating the convergent validity of the scale (i.e. the ‘true’ correlation between the BSES-EBF Scale scores and constructs that tap into the same unobserved behavior) will add another piece of evidence to the validity of the scale [65]. Second, it is our hope that researchers will implement and assess the scale in settings outside of northern Uganda where initiation and continued breastfeeding are common, but rates of exclusive breastfeeding are low, as additional research is required to tests its reliability and predictive effects. Lastly, the use of the current scale can facilitate studies that test the magnitude of effect of exclusive breastfeeding self-efficacy on exclusive breastfeeding practice.

## Conclusions

In summary, this study has demonstrated strong validity and reliability of a modified version of the original BSES-SF scale, the BSES-EBF Scale, which is shorter, more relevant, and valid for use in a setting with low rates of exclusive breastfeeding. Its use will surely enhance our understanding of the correlates of exclusive breastfeeding self-efficacy and exclusive breastfeeding practices globally.

## Additional files

Additional file 1: **Table S1.** Survey module on exclusive breastfeeding self-efficacy as implemented in PostNAPS. Strikethrough indicates items that were ultimately dropped, highlighting refers to items developed. (PDF 60 kb)

Additional file 2: **Figure S1.** Scree plot showing cut-off point for retained scale factors using parallel analysis. (PDF 98 kb)

Additional file 3: **Table S2.** Confirmatory Factor Analysis Modification Indices Output from Mplus showing WITH statements for model re-specification. (PDF 52 kb)

Additional file 4: **Table S3.** Indicators of Validity for Cognitive and Functional sub-scales of the BSES-EBF Scale at 1,3, and 6 months postpartum among Ugandan women (*N* = 239). (DOCX 14 kb)

Additional file 5: **Table S4.** Measures of Discriminant validity: Population correlation estimates of the Breastfeeding Self-Efficacy Scale to Measure Exclusive Breastfeeding with Exclusive Breastfeeding Social Support and Depression Scores using Latent Variable Modeling with model fit indices at 1 month postpartum (*n* = 239) (PDF 91 kb)

## Abbreviations

AIC: Akaike Information Criterion
BF: Breastfeeding
BIC: Bayesian Information Criterion
BSES: Breastfeeding Self-Efficacy Scale
BSES-EBF: Breastfeeding Self-Efficacy Scale to Measure Exclusive Breastfeeding
BSES-SF: Breastfeeding Self-Efficacy Scale – Short Form
CES-D: Centre for Epidemiological Studies-Depression Scale
CFA: Confirmatory Factor Analysis
CFI: Comparative Fit Index
EBF: Exclusive Breastfeeding
EBFSS: Exclusive Breastfeeding Social Support
EFA: Exploratory Factor Analysis
LVM: Latent Variable Modeling
ML: Maximum Likelihood
PostNAPS: Postnatal Nutrition and Psychosocial Health Outcomes Study
PreNAPS: Prenatal Nutrition and Psychosocial Health Outcomes Study
RMSEA: Root Mean Square of Error Approximation
SD: Standard Deviations
SRMR: Standardized Root Mean Square Residual
TLI: Tucker Lewis Index
WRMR: Weighted Root Mean Square Residual

## References

[1] Butte NF, Lopez-Alarcon MG, Garza C (2002). Nutrient adequacy of exclusive breastfeeding for the term infant during the first six months of life.

[2] Victora CG, Bahl R, Barros AJD, França GVA, Horton S, Krasevec J (2016). Breastfeeding in the 21st century: epidemiology, mechanisms, and lifelong effect. Lancet.

[3] Rollins NC, Bhandari N, Hajeebhoy N, Horton S, Lutter CK, Martines JC (2016). Why invest, and what it will take to improve breastfeeding practices?. Lancet.

[4] American Academy of Pediatrics (2012). Breastfeeding and the use of human Milk. Pediatrics.

[5] CDC. Breastfeeding report card. Centers for Disease Control and Prevention. 2016;2017 https://www.cdc.gov/breastfeeding/data/reportcard.htm. Accessed 23 Jun 2017.

[6] Labbok MH (2013). Breastfeeding: population-based perspectives. Pediatr Clin N Am.

[7] WHO. WHO | Exclusive breastfeeding for six months best for babies everywhere. WHO. 2011. https://www.who.int/mediacentre/news/statements/2011/breastfeeding_20110115/en/. Accessed 23 June 2017.

[8] Hansen K (2016). Breastfeeding: a smart investment, and what it will take to improve breastfeeding practices?. Lancet.

[9] WHO/UNICEF. Global Breastfeeding Scorecard, 2017. Tracking Progress for Breastfeeding Policies and Programmes. USA: United Nations Children’s Fund (UNICEF)/World Health Organization (WHO); 2017.

[10] de la Mora A, Russell DW, Dungy CI, Losch M, Dusdieker L (1999). The Iowa infant feeding attitude scale: analysis of reliability and Validity1. J Appl Soc Psychol.

[11] Dennis C-L (2003). The breastfeeding self-efficacy scale: psychometric assessment of the short form. J Obstet Gynecol Neonatal Nurs.

[12] Gewa CA, Oguttu M, Savaglio L. Determinants of early child-feeding practices among HIV-infected and noninfected mothers in rural Kenya. J Human Lactation. 2011;27:239-49. 10.1177/0890334411403930 21788653

[13] Fuller J, White A (1998). The effects of support networks on the choice of infant feeding method. J Am Diet Assoc.

[14] Cattaneo A (2011). Academy of breastfeeding medicine Founder’s lecture 2011: inequalities and inequities in breastfeeding: an international perspective. Breastfeed Med.

[15] McCoach ., Gable RK, Madura J Instrument Development in the Affective Domain: School and Corporate Applications. 3rd edition. Springer New York; 2013.

[16] Bandura A (1977). Self-efficacy: toward a unifying theory of behavioral change. Psychol Rev.

[17] Dennis C (1999). Theoretical underpinnings of breastfeeding confidence: a self-efficacy framework. J Hum Lact.

[18] Blyth R, Creedy DK, Dennis C-L, Moyle W, Pratt J, De Vries SM (2002). Effect of maternal confidence on breastfeeding duration: an application of breastfeeding self-efficacy theory. Birth.

[19] Cleveland AP, McCrone S (2005). Development of the breastfeeding personal efficacy beliefs inventory: a measure of Women’s confidence about breastfeeding. J Nurs Meas.

[20] Hill PD, Humenick SS (1996). Development of the H & H Lactation Scale : nursing research. Nurs Res.

[21] Wells KJ, Thompson NJ, Kloeblen-Tarver AS (2006). Development and psychometric testing of the prenatal breast-feeding self-efficacy scale. Am J Health Behav.

[22] Marinelli KA, Gill SL, Tuthill EL, McGrath JM, Graber M, Cusson RM (2016). Breastfeeding self-efficacy: a critical review of available instruments. J Hum Lact.

[23] UDHS, ICF. Uganda demographic and health survey. Uganda Bureau of Statistics, Kampala Uganda 2011. Uganda Bureau of Statistics (UBOS) and ICF International Inc.; 2012.

[24] Natamba BK, Kilama H, Arbach A, Achan J, Griffiths JK, Young SL. Reliability and validity of an individually focused food insecurity access scale for assessing inadequate access to food among pregnant Ugandan women of mixed HIV status 2015;18:2895–2905. 10.1017/S1368980014001669 PMC10271257 25171462

[25] Widen EM, Collins SM, Khan H, Biribawa C, Acidri D, Achoko W, et al. Food insecurity, but not HIV-infection status, is associated with adverse changes in body composition during lactation in Ugandan women of mixed HIV status. Am J Clin Nutr. 2017:ajcn142513. 10.3945/ajcn.116.142513. 10.3945/ajcn.116.142513 PMC5267304 28052888

[26] Natamba BK, Achan J, Arbach A, Oyok TO, Ghosh S, Mehta S (2014). Reliability and validity of the center for epidemiologic studies-depression scale in screening for depression among HIV-infected and -uninfected pregnant women attending antenatal services in northern Uganda: a cross-sectional study. BMC Psychiatry.

[27] Uganda Bureau of Statistics. The Uganda National Panel Survey 2009/10: Household questionnaire. Kampala; 2010. https://www.ubos.org/onlinefiles/uploads/ubos/pdf%20documents/UNPS%2009_10_ManualofInstructions.pdf. Accessed 25 May 2017.

[28] Radloff LS (1977). The CES-D scale: a self-report depression scale for research in the general population. Appl Psychol Meas.

[29] Broadhead WE, Gehlbach SH, De Gruy FV, Kaplan BH (1988). The Duke-UNC functional social support questionnaire: measurement of social support in family medicine patients. Med Care.

[30] Boateng GO, Martin SL, Collins S, Natamba BK, Young SL (2018). Measuring exclusive breastfeeding social support: scale development and validation. Maternal & Child Nutrition.

[31] Wutke K, Dennis C-L (2007). The reliability and validity of the polish version of the breastfeeding self-efficacy scale-short form: translation and psychometric assessment. Int J Nurs Stud.

[32] Zubaran C, Foresti K, Schumacher M, Thorell MR, Amoretti A, Müller L (2010). The Portuguese version of the breastfeeding self-efficacy scale—short form. J Hum Lact.

[33] McCarter-Spaulding DE, Dennis C-L (2010). Psychometric testing of the breastfeeding self-efficacy scale-short form in a sample of black women in the United States. Res Nurs Health..

[34] Oliver-Roig A, M-L D’A-G, García-García B, Silva-Tubio J-R, Richart-Martínez M, Dennis C-L (2012). The Spanish version of the breastfeeding self-efficacy scale-short form: reliability and validity assessment. Int J Nurs Stud.

[35] Aluş Tokat M, Okumuş H, Dennis C-L (2010). Translation and psychometric assessment of the breast-feeding self-efficacy scale—short form among pregnant and postnatal women in Turkey. Midwifery.

[36] Dodt RCM, Ximenes LB, Almeida PC, MOB O, Dennis C-L (2012). Psychometric and maternal sociodemographic assessment of the breastfeeding self-efficacy scale - short form in a brazilian sample. J Nurs Educ Pract.

[37] Thurstone L (1947). Multiple-factor analysis.

[38] Raykov T, Marcoulides GA (2011). Introduction to psychometric theory.

[39] Raykov T, Marcoulides GA (2011). Classical item analysis using latent variable modeling: a note on a direct evaluation procedure. Struct Equ Model Multidiscip J.

[40] Boateng GO, Neilands TB, Frongillo E, Melgar-Quiñonez HR, Young SL (2018). Best practices for developing and validating scales for health. Social, and Behavioral Research: A Primer.

[41] Edwards A (1957). Technique of attitude scale construction.

[42] Raykov T (2015). Scale construction and development (lecture notes).

[43] Cattell RB (1966). The scree test for the number of factors. Multivar Behav Res.

[44] Guttman L (1954). Some necessary conditions for common-factor analysis. Psychometrika.

[45] Kaiser HF (1960). The application of electronic computers to factor analysis. Educ Psychol Meas.

[46] Nunnally JC (1978). Pyschometric theory.

[47] Muthén L, Muthén B (2015). Mplus User’s guide. Seventh.

[48] Horn JL (1965). A rationale and test for the number of factors in factor analysis. Psychometrika.

[49] Bentler PM (1990). Comparative fit indexes in structural models. Psychol Bull.

[50] Bentler PM, Bonett DG (1980). Significance tests and goodness of fit in the analysis of covariance structures. Psychol Bull.

[51] Browne MW, Cudeck R, Bollen KA, lONG JS (1993). Alternative ways of assessing model fit. testing structural equation models (PP. 136–162).

[52] Hu L, Bentler PM (1999). Cutoff criteria for fit indexes in covariance structure analysis: conventional criteria versus new alternatives. Struct Equ Model Multidiscip J.

[53] Kline RB (2010). Principles and practice of structural equation modeling.

[54] Tucker LR, Lewis C (1973). A reliability coefficient for maximum likelihood factor analysis. Psychometrika.

[55] Brown T (2014). Confirmatory factor analysis for applied research.

[56] Bollen KA (1989). Structural equations with latent variables.

[57] Byrne BM. Structural equation modeling with Mplus: Basic concepts, applications, and programming. New York: Routledge, Taylor & Francis Group; 2013.

[58] Porta M (2008). A dictionary of epidemiology.

[59] Cronbach LJ (1951). Coefficient alpha and the internal structure of tests. Psychometrika.

[60] DeVellis RF (2012). Scale development: theory and application.

[61] Bernstein I, Nunnally JC (1994). Pyschometric theory.

[62] Churchill GA (1979). A paradigm for developing better measures of marketing constructs. J Mark Res.

[63] Dennis C-L, Faux S (1999). Development and psychometric testing of the breastfeeding self-efficacy scale. Res Nurs Health.

[64] Nommsen-Rivers LA, Dewey KG (2009). Development and validation of the infant feeding intentions scale. Matern Child Health J.

[65] Campbell DT, Fiske DW (1959). Convergent and discriminant validity by the multitrait-multimethod matrix. Psychol Bull.

[66] Coates R, Ayers S, De VR (2014). Women’s experiences of postnatal distress: a qualitative study. BMC Pregnancy Childbirth.

[67] Zubaran C, Foresti K (2013). The correlation between breastfeeding self-efficacy and maternal postpartum depression in southern Brazil. Sex Reprod Healthc.

