# Supplementary material for: Adaptation and psychometric evaluation of the breastfeeding self-efficacy scale to assess exclusive breastfeeding
Source: BMC Pregnancy Childbirth. 2019 Feb 18;19:73. doi: 10.1186/s12884-019-2217-7 (PMC6380059; doi:10.1186/s12884-019-2217-7)
Supplement: Supplementary file 1 — Table S1. Survey module on exclusive breastfeeding self-efficacy as implemented in PostNAPS. Strikethrough indicates items that were ultimately dropped, highlighting refers to items developed. (PDF 60 kb) [file 12884_2019_2217_MOESM1_ESM.pdf]

Table S1.

Prompt: "I have some questions about how you feel about exclusively breastfeeding your baby. Tell me how you would rank your confidence on a scale of 1 to 5 for each situation. There is no right or wrong answer."			
1 Month Label	3 Month Label	Variable Label	Items
D2A9	D2A10	Enough Milk	I can always determine that my baby is getting enough milk*
~~D2B9~~	~~D2B10~~	~~Challenging Tasks~~	I can always successfully cope with exclusive breastfeeding like I have with other challenging tasks*
D2C9	D2C10	EBF Milk	I can always give my baby only breast milk without using animal milk, formula, or other liquids or foods as a supplement**
~~D2D9~~	~~D2D10~~	~~Baby Latched~~	I can always ensure that my baby is properly latched on for the whole feeding*
~~D2E9~~	~~D2E10~~	~~Manage BF~~	I can always manage the breastfeeding situation to my satisfaction*
~~D2F9~~	~~D2F10~~	~~BF Baby crying~~	I can always manage to breastfeed even if my baby is crying*
D2G9	D2G10	Continue EBF	I can continue exclusively breastfeeding for as long as I want**
~~D2H9~~	~~D2H10~~	~~BF Family members~~	I can always comfortably breastfeed with my family members present*
D2I9	D2I10	Satisfy BF	I can always be satisfied with my breastfeeding experience*
D2J9	D2J10	Deal BF	I can always deal with the fact that breastfeeding can be time consuming*
~~D2K9~~	~~D2K10~~	~~Finish feeding~~	I can always finish feeding my baby on one breast before switching to the other breast*
D2L9	D2L10	BF Every feeding	I can always continue to breastfeed my baby for every feeding*
D2M9	D2M10	Keep Bf demands	I can always manage to keep up with my baby's breastfeeding demands*
~~D2N9~~	~~D2N10~~	~~Finished BF~~	I can always tell when my baby is finished breastfeeding*
~~D209~~	~~D2010~~	~~BF Baby hunger~~	I can always make enough breast milk to satisfy my baby's hunger***
~~D2P9~~	~~D2P10~~	~~Quality breastmilk~~	I can always make good quality breast milk that has everything that my baby needs to grow well and be healthy***
~~D2Q9~~	~~D2Q10~~	~~Make decisions~~	I can always make decisions about how my baby is being fed***
D2R9	D2R10	EBF Liquid	I can always exclusively breastfeed without my baby receiving even a drop of water or any other liquid***

Prompt: "I have some questions about how you feel about exclusively breastfeeding your baby. Tell me how you would rank your confidence on a scale of 1 to 5 for each situation. There is no right or wrong answer."			
1 Month Label	3 Month Label	Variable Label	Items
D2S9	D2S10	Stop Other foods	I can always stop someone from trying to feed my baby liquids or foods other than breast milk, including purchased baby foods (e.g. infant formula, milk, porridge, juice, tea [whatever is given]), before 6 months of age***

Notes: *Questions directly adapted from the Breastfeeding Self-Efficacy Scale-Short Form, **Questions modified from the Breastfeeding Self-Efficacy Scale-Short Form, ***Newly developed questions; Response categories are 1=Not at all confident, 2=Not very confident, 3=Sometimes confident, 4=Confident, 5=Very confident; Items struck through were dropped after item correlations and factor analysis.
