# Supplementary figures and images for: Adaptation and psychometric evaluation of the breastfeeding self-efficacy scale to assess exclusive breastfeeding
Source: BMC Pregnancy Childbirth. 2019 Feb 18;19:73. doi: 10.1186/s12884-019-2217-7 (PMC6380059; doi:10.1186/s12884-019-2217-7)

**Fig S1.**

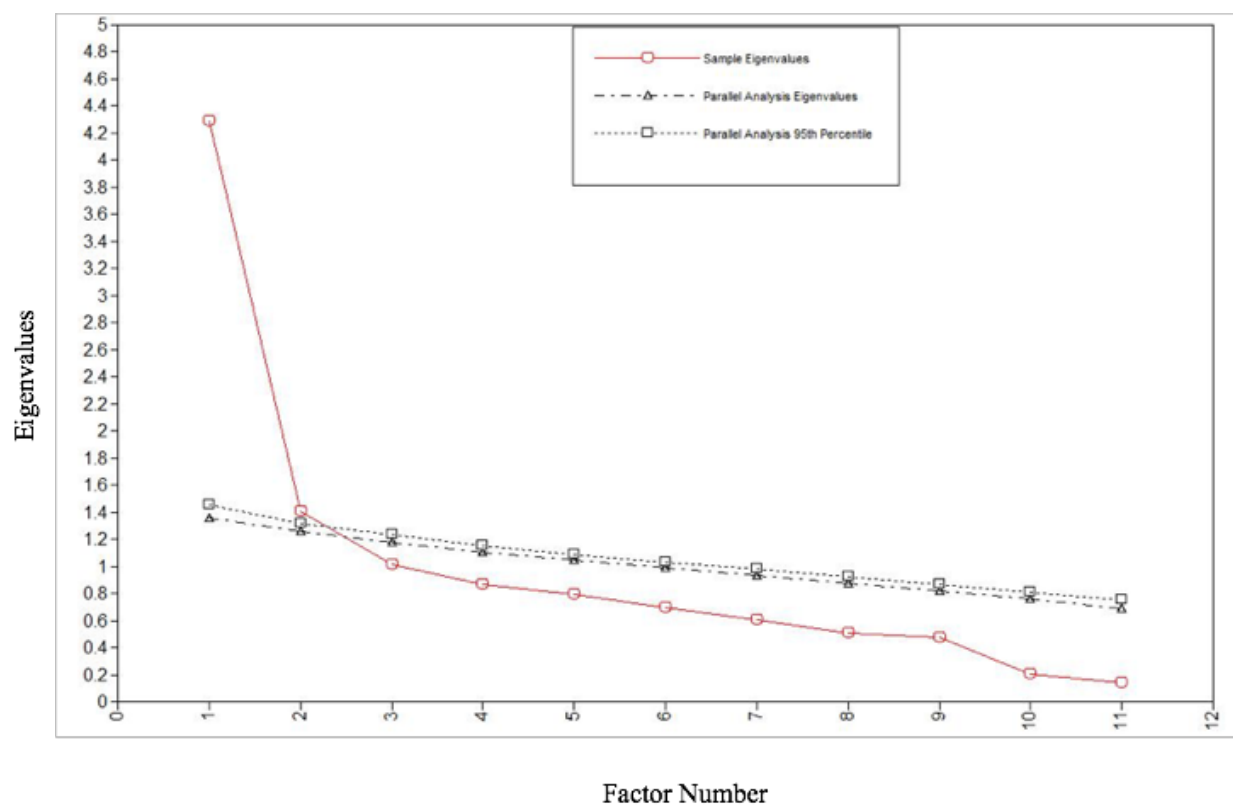

Supplement: Supplementary file 2 — Figure S1. Scree plot showing cut-off point for retained scale factors using parallel analysis. (PDF 98 kb) [file 12884_2019_2217_MOESM2_ESM.pdf]
