# Supplementary material for: Adaptation and psychometric evaluation of the breastfeeding self-efficacy scale to assess exclusive breastfeeding
Source: BMC Pregnancy Childbirth. 2019 Feb 18;19:73. doi: 10.1186/s12884-019-2217-7 (PMC6380059; doi:10.1186/s12884-019-2217-7)
Supplement: Supplementary file 3 — Table S2. Confirmatory Factor Analysis Modification Indices Output from Mplus showing WITH statements for model re-specification. (PDF 52 kb) [file 12884_2019_2217_MOESM3_ESM.pdf]

Table S2.

	M.I.	E.P.C.	Std E.P.C.	StdYX E.P.C
**WITH STATEMENTS**				
D2L10 WITH D2M10	19.49	0.33	0.33	1.73
D2S10 WITH D2R10	13.94	0.23	0.23	0.56

Notes: M.I.=Modification indices; E.P.C.=expected unstandardized parameter change; Std E.P.C.=expected standardized parameter change; Std E.P.C.=fully standardized loading; D2S10 = Stop Other foods; D2R10=EBF Liquid; D2L10=BF Every feeding; D2M10=Keep Bf demands
