# Supplementary material for: Adaptation and psychometric evaluation of the breastfeeding self-efficacy scale to assess exclusive breastfeeding
Source: BMC Pregnancy Childbirth. 2019 Feb 18;19:73. doi: 10.1186/s12884-019-2217-7 (PMC6380059; doi:10.1186/s12884-019-2217-7)
Supplement: Supplementary file 4 — Table S3. Indicators of Validity for Cognitive and Functional sub-scales of the BSES-EBF Scale at 1,3, and 6 months postpartum among Ugandan women (N = 239). (DOCX 14 kb) [file 12884_2019_2217_MOESM4_ESM.docx]

| **Additional file 4: Table S3**. Indicators of Validity for Cognitive and Functional sub scales of BSES-EBF at 1, 3, and 6 months postpartum among Ugandan Women (N=239) | | | |
| --- | --- | --- | --- |
| Exclusive Breastfeeding at | 1 Month | 3 Months | 6 Months |
| **Predictive Validity** |  |  |  |
| Cognitive BSES-EBF  @1 month | **OR**=1.06, 95%CI: 1.01, 1.11; *p*=0.014 | **OR**=1.08, 95%CI: 1.03,1.12; *p*=0.001 | **OR**=1.07, 95%CI:0.99,1.15; *p*=0.06 |
| Functional BSES-EBF  @1 month | **OR**=1.06, 95%CI: 1.02, 1.10; *p*=0.005 | **OR**=1.02, 95%CI:0.98,1.06; *p*=0.289 | **OR**=1.08,95%CI:0.99,1.16; *p*=0.07 |
| Cognitive BSES-EBF @3 Months |  | **OR**=1.15, 95%CI: 1.08,1.21; *p*=0.000 | **OR**=1.19, 95%CI: 1.09, 1.29; *p*<0.001 |
| Functional BSES-EBF @3 Months |  | **OR**=1.07, 95%CI:1.03,1.12; *p*=0.001 | **OR**=1.07, 95%CI:0.99,1.44; *p*=0.08 |
| **Known group comparison** | **Cognitive EBFSE** | **Functional EBFSE** |  |
| Primiparae vs. Multiparae | 12.96 vs 13.67, t=-1.12, *p*=0.26 | 16.51 vs. 17.36, t=-1.28, *p*=0.20 |  |
| Correct Breastfeeding knowledge (No vs. Yes) | 12.24 vs. 14.42, t=-4.19, *p*=0.001*** | 16.81 vs. 17.41, t=-1.07, *p*=0.28 |  |
| Notes: BSES-EBF=Breastfeeding Self Efficacy Scale to Measure Exclusive Breastfeeding; Cognitive = 4 items; Functional=5 items; Primiparae=first time birth with no breastfeeding experience; Multiparae=have more than one child with breastfeeding experience; OR=Odds Ratios; CI: Confidence Intervals. | | | |
