# Supplementary material for: Adaptation and psychometric evaluation of the breastfeeding self-efficacy scale to assess exclusive breastfeeding
Source: BMC Pregnancy Childbirth. 2019 Feb 18;19:73. doi: 10.1186/s12884-019-2217-7 (PMC6380059; doi:10.1186/s12884-019-2217-7)
Supplement: Supplementary file 5 — Table S4. Measures of Discriminant validity: Population correlation estimates of the Breastfeeding Self-Efficacy Scale to Measure Exclusive Breastfeeding with Exclusive Breastfeeding Social Support and Depression Scores using Latent Variable Modeling with model fit indices at 1 month postpartum (n = 239) (PDF 91 kb) [file 12884_2019_2217_MOESM5_ESM.pdf]

Table S4.

Measures	$\chi 2$	*df*	RMSEA	CFI	TLI	WRMR	ρ	[95%CI]
**BSES-EBF and EBFSS**								
Cognitive BSES-EBF and Instrumental EBFSS	24.877*	13	0.06	0.99	0.99	0.61	0.09	[-0.05,0.23]
Cognitive BSES-EBF and Informational EBFSS	53.052*	26	0.07	0.99	0.98	0.80	0.23***	[0.10, 0.36]
Cognitive BSES-EBF and Emotional EBFSS	119.892*	53	0.07	0.98	0.97	0.94	0.28***	[0.16, 0.40]
Functional BSES-EBF and Instrumental EBFSS	77.183*	19	0.11	0.98	0.97	1.00	0.30***	[0.18, 0.43]
Functional BSES-EBF and Informational EBFSS	98.200*	34	0.09	0.97	0.97	0.99	0.39***	[0.28, 0.50]
Functional BSES-EBF and Emotional EBFSS	268.087*	64	0.12	0.94	0.94	1.37	0.47***	[0.38, 0.57]
**BSES-EBF and CESD Score**								
Cognitive BSES-EBF and Depression	806.935*	251	0.09	0.87	0.85	1.60	-0.09	[-0.24, 0.05]
Functional BSES-EBF and Depression	1098.63*	274	0.11	0.81	0.81	1.90	-0.14**	[-0.28, -0.01]

Notes: **BSES-EBF=** Breastfeeding Self-Efficacy Scale to Measure Exclusive Breastfeeding; **EBFSS**=Exclusive Breastfeeding Social Support; **$\chi 2$** = chi-square goodness of fit statistic; $\chi 2$: df ratio of less than 3:1; ***df***=degrees of freedom; **RMSEA (≤0.08)** = Root Mean Square Error of Approximation; **CFI (≥0.95)**=Comparative Fit Index; **TLI (≥0.95)**=Tucker Lewis Index; **SRMR(≤0.08)** =Standardized Square Root Mean Residual; **WRMR** (≤1.0)=Weighted Root Mean Square Residual; ρ = Rho=population correlation between two latent variables; CI: Confidence Interval; *All goodness-of-fit tests were statistically significant at $p<0.001$;**discriminant correlation estimate significant at p<0.5; ***discriminant correlation estimate significant at $p<0.0001$
